# Bioenergetic and Antiapoptotic Properties of Mitochondria from Cultured Human Prostate Cancer Cell Lines PC-3, DU145 and LNCaP

**DOI:** 10.1371/journal.pone.0072078

**Published:** 2013-08-08

**Authors:** Alexander Panov, Zulfiya Orynbayeva

**Affiliations:** 1 Institute of Molecular Biology and Biophysics, Siberian Division of the Russian Academy of Medical Sciences, Novosibirsk, Russia; 2 Department of Surgery, Drexel University College of Medicine, Philadelphia, Pennsylvania, United States of America; Southern Illinois University School of Medicine, United States of America

## Abstract

The purpose of this work was to reveal the metabolic features of mitochondria that might be essential for inhibition of apoptotic potential in prostate cancer cells. We studied mitochondria isolated from normal prostate epithelial cells (PrEC), metastatic prostate cancer cell lines LNCaP, PC-3, DU145; and non-prostate cancer cells - human fibrosarcoma HT1080 cells; and normal human lymphoblastoid cells. PrEC cells contained 2 to 4 times less mitochondria per gram of cells than the three PC cell lines. Respiratory activities of PrEC cell mitochondria were 5-20-fold lower than PC mitochondria, depending on substrates and the metabolic state, due to lower content and lower activity of the respiratory enzyme complexes. Mitochondria from the three metastatic prostate cancer cell lines revealed several features that are distinctive only to these cells: low affinity of Complex I for NADH, 20-30 mV higher electrical membrane potential (ΔΨ). Unprotected with cyclosporine A (CsA) the PC-3 mitochondria required 4 times more Ca^2+^ to open the permeability transition pore (mPTP) when compared with the PrEC mitochondria, and they did not undergo swelling even in the presence of alamethicin, a large pore forming antibiotic. In the presence of CsA, the PC-3 mitochondria did not open spontaneously the mPTP. We conclude that the low apoptotic potential of the metastatic PC cells may arise from inhibition of the Ca^2+^-dependent permeability transition due to a very high ΔΨ and higher capacity to sequester Ca^2+^. We suggest that due to the high ΔΨ, mitochondrial metabolism of the metastatic prostate cancer cells is predominantly based on utilization of glutamate and glutamine, which may promote development of cachexia.

## Introduction

Prostate cancer is the major cause of male cancer death in the age range of 55-74, and above age 75 it is the second greatest cause of death in North American men after lung and bronchus cancer [1,2]. Essentially all men with advanced disease, who went through androgen deprivation therapies, eventually die because of development of androgen-independent metastatic prostate cancer [1,3,4]. The high level of mortality from prostate cancer is associated with active proliferation of the prostate adenocarcinoma which disseminates to distant organs with preferences to the bone tissue [5]. There is a large body of data, which indicates that progression of both primary and metastatic prostatic tumors is determined by the loss of the cell’s apoptotic potential [6–8].

The participation of mitochondria in apoptosis has been substantiated by a large number of reports describing proapoptotic mitochondrial alterations, such as production of reactive oxygen species (ROS), depletion of ATP, and induction of the mitochondrial permeability transition pore (mPTP) [9–11]. It has been shown that Bcl-2 and other apoptosis-regulating proteins of this family are located at the mitochondrial junction sites of the inner and outer membranes or the intermembrane space and regulate apoptosis through their effects on mitochondrial permeability transition [12–15]. Studies on relationships between induction of apoptosis in prostate cancer cells and expression of Bcl-2 and Bax-related proteins gave contradictory results [16–21], and the data suggest that Bcl-2, Bcl-xL and some other apoptosis-related proteins are not important for induction of apoptosis in prostate cancer cells [18,19,22–24]. On the other hand, opening of the permeability transition pore directly depends on mitochondrial properties such as electrical membrane potential (ΔΨ), production of ROS [25], and respiratory activity [26–28]. Therefore, it is important to understand biochemical and physiological aspects of mitochondrial functionality as a central gate-keeper in the inability of prostate cancer cells to commit to programmed cell death.

While there are many reports on apoptosis induction in prostate cells via modulating mitochondrial metabolism [29–31], overall not much is known about the bioenergetics and mitochondrial functions of normal or cancerous prostatic cells, except the differences in their metabolisms of citric acid [32] and mitochondrial L-lactate [33]. It has been shown that unlike most malignant tissues, prostate tumor cells are characterized by a low rate of glycolysis and glucose uptake [34,35], and by preferential uptake of fatty acids over glucose [36]. The high biochemical plasticity of prostate cancer cells helps them to adapt their metabolism to typical tumor hypoxic condition [37]. However, in many of these studies on mitochondrial metabolism in prostate cancer cells, the authors used antibiotics [29,31,36–38]. It is known that aminoglycoside antibiotics (streptomycin, gentamicin) are mitotoxic [39–41]. We have established that mitochondria isolated from prostate cancer cells, human lymphoblastoid cells and hepatocytes grown in the presence of streptomycin do not respire on any substrates. Thus cells in the cultures containing antibiotics do not maintain aerobic metabolism, and glycolysis is the only source of ATP. Therefore many conclusions obtained on cell cultures with antibiotics have to be regarded with caution.

Early studies on the ultramicroscopic structure of normal and cancerous prostate cells have indicated that prostate cancer cells show a striking increase in the number and pleomorphism of mitochondria [42]. This separates prostate cancer from other cancer types where malignant transformation is usually accompanied by a significant decrease in the cell’s mitochondria [43].

In the normal prostate, epithelial cells secrete a high level of citrate probably due to their relative inability to oxidize citrate via the Krebs cycle [32,44]. Prostate citrate levels increase even further in benign hyperplasia of prostate, but drop sharply during development of the prostate cancer, presumably because cancer mitochondria acquire the capacity to oxidize citrate [32]. This metabolic property of prostate cancer mitochondria is the antithesis of that observed for many fast growing cancers, in which the Krebs cycle switches from citrate utilization to citrate production, resulting in increased cholesterol production [45].

The contribution of energy metabolism in cancer development and progression has been reported in a number of works [44,46,47]. It is believed that to make anticancer therapy cancer-specific, the bioenergetic metabolic features of each tumor type have to first be elucidated. The alterations in bioenergetic functions of human prostate cancer LNCaP, DU145 and PC-3 cells have been shown to be related to mitochondrial dysfunctions [38], while the detailed mechanisms of the mitochondrial pathology remain uncertain. The purpose of this study was to elucidate those metabolic features of mitochondria that might contribute to the inhibition of apoptosis in prostate cancer cells. Because of known extraordinary heterogeneity of prostate cancer we examined the mitochondrial bioenergetic properties of three prostate cancer cell lines, namely PC-3, LNCaP and DU145, differing in their origin, tumorigenicity, response to androgens, and proliferation rates [38,43,48,49]. For comparison, we studied mitochondria from the cultured normal human prostate epithelial cells (PrEC). All these cell lines are virtually unstudied in terms of mitochondrial functions. For the sake of comparison, we studied also a non-prostate human fibrosarcoma cell line HT1080. The EBV-transformed normal human lymphoblastoid cells (HLB) served as a reference to how mitochondria from cultured normal cells respond to Ca^2+^ loads and cyclosporine A.

This is the first investigation into the bioenergetic properties and the Ca^2+^-dependent permeability transition of isolated mitochondria from the established prostate cancer cell lines and normal human PrEC cells. We report here that mitochondria from the three metastatic prostate cancer cell lines have a number of distinct metabolic features: a 20 to 30 mV higher electrical membrane potential (ΔΨ), low affinity of the Complex I to NADH, higher resistance to Ca^2+^ loads, and an unusual response to cyclosporine A and the pore forming antibiotic Alamethicin, when compared with the PrEC and normal HLB mitochondria. The observed features of prostate cancer mitochondria may protect the prostate cancer cells from apoptosis by direct and indirect inhibition of mitochondrial permeability transition.

## Results

### Mitochondrial yields

**Figure 1:**
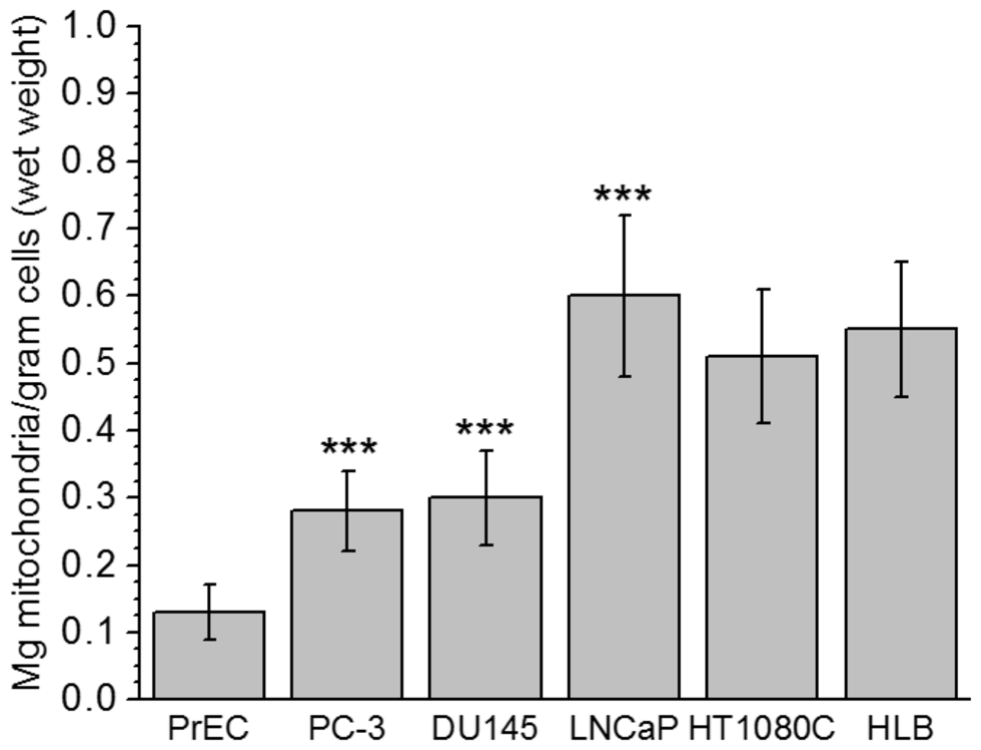
Mitochondrial yields from cultured normal human prostate PrEC cells, prostate cancer cells PC-3, DU145, LNCaP, human fibrosarcoma cells HT1080C, and human lymphoblastoid cells (HLB). Mitochondria were prepared as described in Methods. The results are presented as Mean ± SE, n = 5-7 (separate isolations from cells). Values are expressed as mg mitochondrial protein per 1 gram of wet cells. Statistics: ** *p* < 0.05; *** *p* < 0.001. Values for prostate cancer cells PC-3, DU145 and LNCaP were compared with normal prostate cells PrEC.

Figure 1 shows the yields of mitochondria isolated from the cells under the study. In comparison with the PC-3, DU145 and LNCaP prostate cancer cells, normal prostate PrEC cells yielded correspondingly 2.1, 2.3 and 4.6 times less mitochondria per gram of cells. The highest yields among the prostate cancer cells were obtained with LNCaP cells, and also with fibrosarcoma cells (HT1080C) and normal human lymphoblastoid cells (HLB). The yields were quite consistent for a given cell line, but varied between the cell lines [50].

### Respiratory activities of mitochondria isolated from the cultured cells

**Figure 2:**
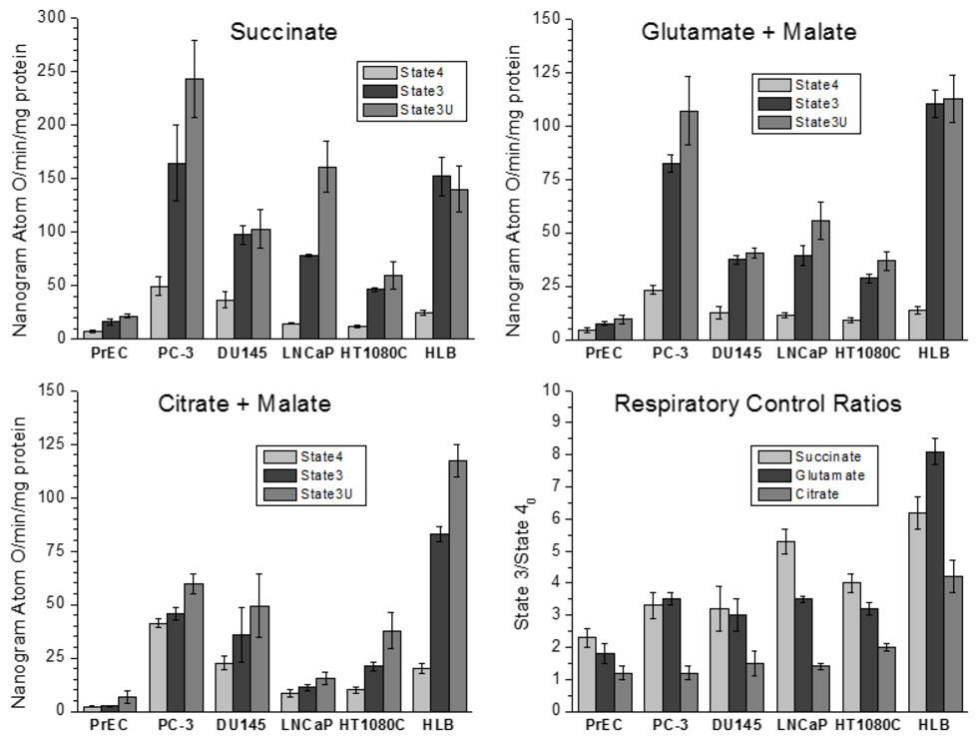
Respiratory activities and respiratory control ratios of mitochondria isolated from normal human prostate PrEC cells, human lymphoblastoid cells (HLB), prostate cancer cells PC-3, DU145, LNCaP, and human fibrosarcoma cells HT1080C. **Incubation conditions** are described in Methods. **Substrates**: succinate 10 mM; glutamate 10 mM + malate 2 mM; citrate 10 mM + malate 2 mM. Oxidative phosphorylation (State 3) respiration was stimulated by addition of 150 µM ADP; uncoupled respiration (State 3U) was stimulated by addition of 0.5 µM cyanide-*m*-chlorophenylhydrazone (CCCP). Respiratory controls were calculated as the ratios of the State 3 respiration rate to the respiration rate in State 4_0_ (before addition of ADP).

Figure 2 shows respiratory activities of the mitochondria in various metabolic states. Because of the limited yields of mitochondria from the cultured cells, particularly from the normal PrEC cells, we restricted the selection of substrates to succinate, glutamate + malate, and citrate + malate. Citrate was selected because of the striking differences in the citrate metabolism in normal and malignant prostate tissues (32,44). Succinate oxidation is an alternative to the NAD-dependent substrates source of electrons. Oxidation of glutamate + malate provides electrons for Complex I, but also may reflect functioning of the Krebs cycle in the split mode. In addition, in the preliminary experiments we have found that prostate cancer mitochondria oxidized glutamate + malate at significantly higher rates than pyruvate + malate.

With succinate and glutamate + malate, the respiratory activities of mitochondria from the three PC cell types under study were manifold higher in all metabolic states, when compared with the mitochondria from normal PrEC cells (Figure 2).

To some extent, the efficiency of the mitochondrial oxidative phosphorylation can be evaluated by the respiratory control ratios (RCR), which are calculated as the ratio of the respiratory rate in State 3 (oxidative phosphorylation) to the respiratory rate in State 4 (resting respiration). However, absolute rates respiration at the State 4 and State 3, are also very important for understanding the mitochondrial energetics. Generally, in mitochondria from normal tissues isolated in the presence of bovine serum albumin, the RCR values are higher with the NAD-dependent substrates than with succinate [51].

Overall, the data presented in Figure 2 clearly demonstrate that normal PrEC mitochondria differ significantly from the prostate cancer cells mitochondria, as well as from the normal and malignant non-prostate cells. Figures 1 and 2 show that cancer transformation of normal prostate tissue cells was accompanied by several-fold increase in the content of mitochondria per cell and the many-fold increase in the mitochondrial respiratory activity.

When normal or prostate cancer cell mitochondria oxidized succinate, addition of ADP or an uncoupler (CCCP) produced respiratory rates higher than the corresponding rates for the NAD-dependent substrates (Figure 2). Thus, the low rates of the State 3 oxidation of glutamate and citrate in cancer mitochondria were not caused by low activity of the ATP/ADP carrier, ATP synthase, or activities of Complexes III and IV, but rather, by low activity of Complex I (NADH dehydrogenase).

### Electrical membrane potentials of mitochondria from the prostate and non-prostate cells

High mitochondrial membrane potentials in carcinoma cells, including prostate cancer cells, when compared to normal epithelial cells, have been reported in several studies [52]. However, most of these data were obtained with fluorescent cationic dyes (Rhodamine 123, JC-1, etc.) that give only a qualitative evaluation of ΔΨ, and, sometimes, erroneous results. The pitfalls of the fluorescent methods for evaluation of the mitochondrial energization in cells have been discussed in the literature [53,54]. With isolated mitochondria, we used a TPP^+^-sensitive electrode that allows quantitatively evaluate the ΔΨ values for membrane potentials higher than -100 mV [55].

**Figure 3:**
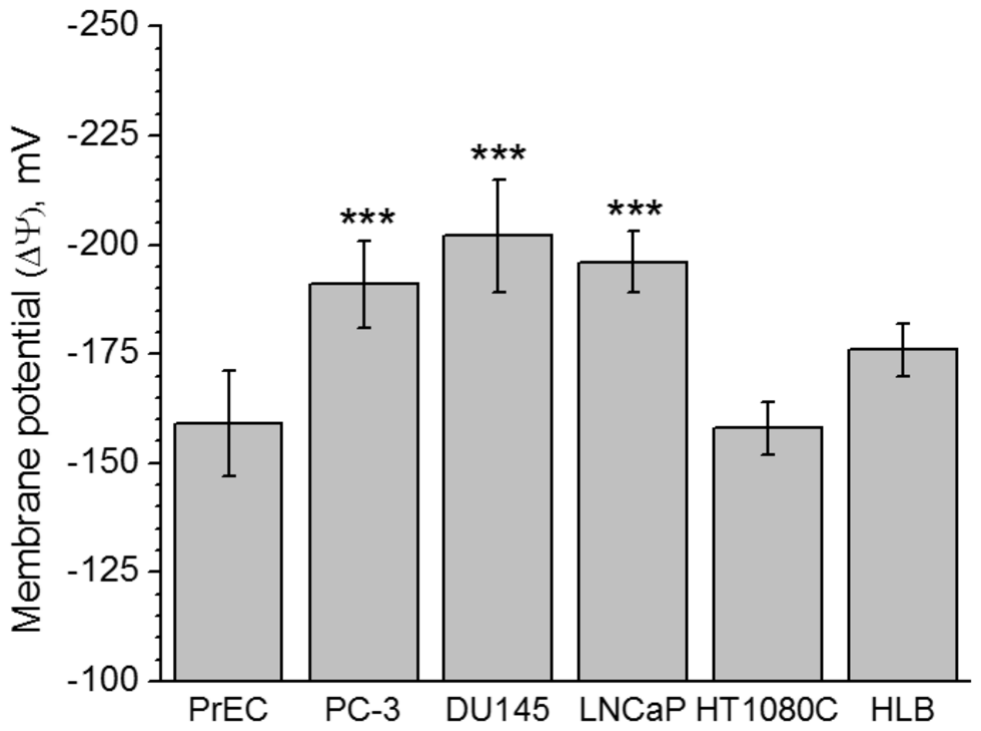
Membrane potential (ΔΨ) of mitochondria from cultured normal human prostate PrEC cells, prostate cancer cells PC-3, DU145, LNCaP, human fibrosarcoma cells HT1080C, and HLB oxidizing succinate in the metabolic State 4. Incubation conditions and calculation of ΔΨ as -mV are described in Methods. Values are expressed as Mean (-mV) ± SE. Statistics: ** *p* < 0.05; *** *p* < 0.001. Values for prostate cancer cells PC-3, DU145 and LNCaP were compared with normal prostate cells PrEC.

Figure 3 reports the ΔΨ values for the mitochondria isolated from the cell lines under study. These values were calculated using corrections for the binding of TPP^+^ to the inner membrane and matrix (IBC, inner binding constant) estimated according to [56] and assuming the matrix volume of 1 µl per 1 mg of mitochondrial protein. Figure 3 shows that the ΔΨ values for the prostate cancer mitochondria were 20 to 30 mV higher than those estimated for PrEC cells, and for the non-prostate cancer HT1080C cells, for which the fluorescent dye method showed higher than normal membrane potential [52]. Importantly, high ΔΨ in the prostate cancer (PC) cell mitochondria was not observed in the cells, which were harvested from the culture flasks that reached about 80-90% confluence. Mitochondria from the almost confluent cultures of PC cells had ΔΨ below -150 mV. Qualitatively, similar results were obtained with the cultured PC-3 cells at 60% and 90% confluence, when the cells were stained with the mitochondria-specific fluorescent dyes with different affinity for energized mitochondria (data are not shown).

### Kinetic properties of Complex I in the submitochondrial particles (SMP)

**Figure 4:**
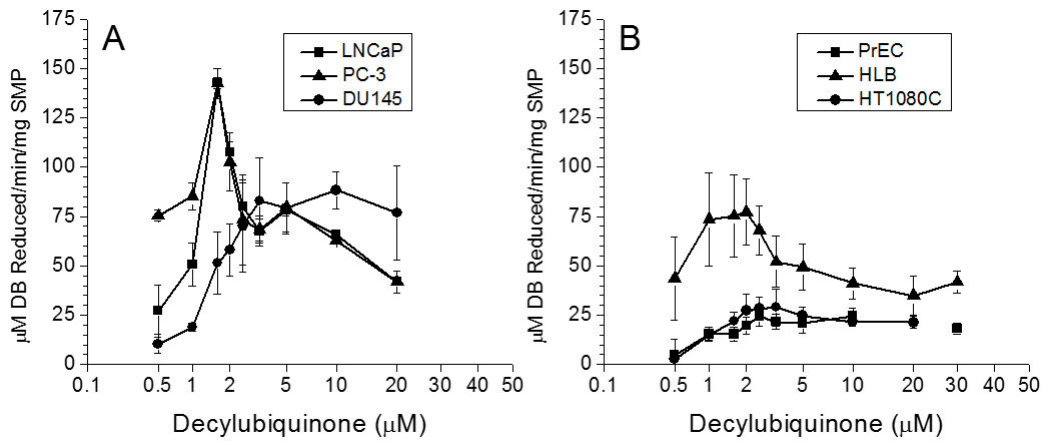
Dependence of Complex I activity on concentration of Decylubiquinone (DB) in submitochondrial particles from normal human PrEC and HLB cells, human prostate cancer cell lines PC-3, DU145, LNCaP and human fibrobsarcoma HT1080 cells. Incubation conditions are described in Methods. SMP (0.15 mg) were incubated with various concentrations of DB for 5 min at 30^o^C; the reaction was started by addition of 1 mM NADH. Panel **A**: the rate of DB reduction by SMP from LNCaP (■), PC-3 (●), and DU145 (▲) cells. Panel **B**: the rate of DB reduction by SMP from PrEC (■), HLB (▲), and HFS (●) cells.

**Figure 5:**
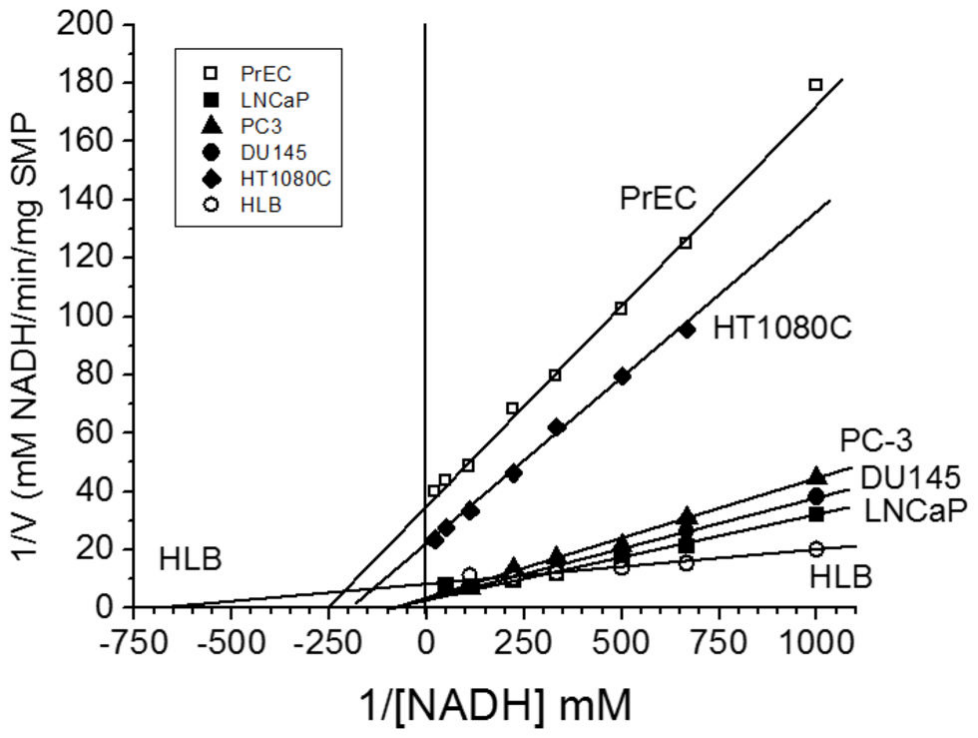
Determination of the Michaelis constants for NADH in the submitochondrial particles from normal human PrEC and HLB cells, human prostate cancer cell lines PC-3, DU145, LNCaP and human fibrobsarcoma HT1080 cells. Incubation conditions as in Figure 2.

To find out why the isolated PC cell mitochondria showed relatively low activities with the NAD substrate (glutamate + malate, citrate + malate) as compared with succinate, we studied the kinetic properties of Complex I. The assay of Complex I involves NADH as the electron donor and an appropriate artificial electron acceptor. So far, 6-Decyl ubiquinone (DB) is the best and most commonly used electron acceptor [57]. The NADH dehydrogenase complex consists of 46 subunits that are organized into L-shaped structure, with the hydrophobic domain embedded into the inner membrane, and the hydrophilic “arm” protruding into the matrix space, and which has binding sites for NADH [58]. The hydrophobic domain encompasses all 7 mitochondrial DNA (mtDNA) encoded subunits that include the coenzyme Q binding domain of the Complex I in cancerous and non-cancerous SMP. First, we estimated the optimal concentration of DB for each type of mitochondria. For this we titrated DB in the presence of excess of NADH (Figure 4 A, B), and then titrated NADH in the presence of the optimal concentration of DB for determination of the Michaelis constants (Km) for NADH (Figure 5).

**Figure 6:**
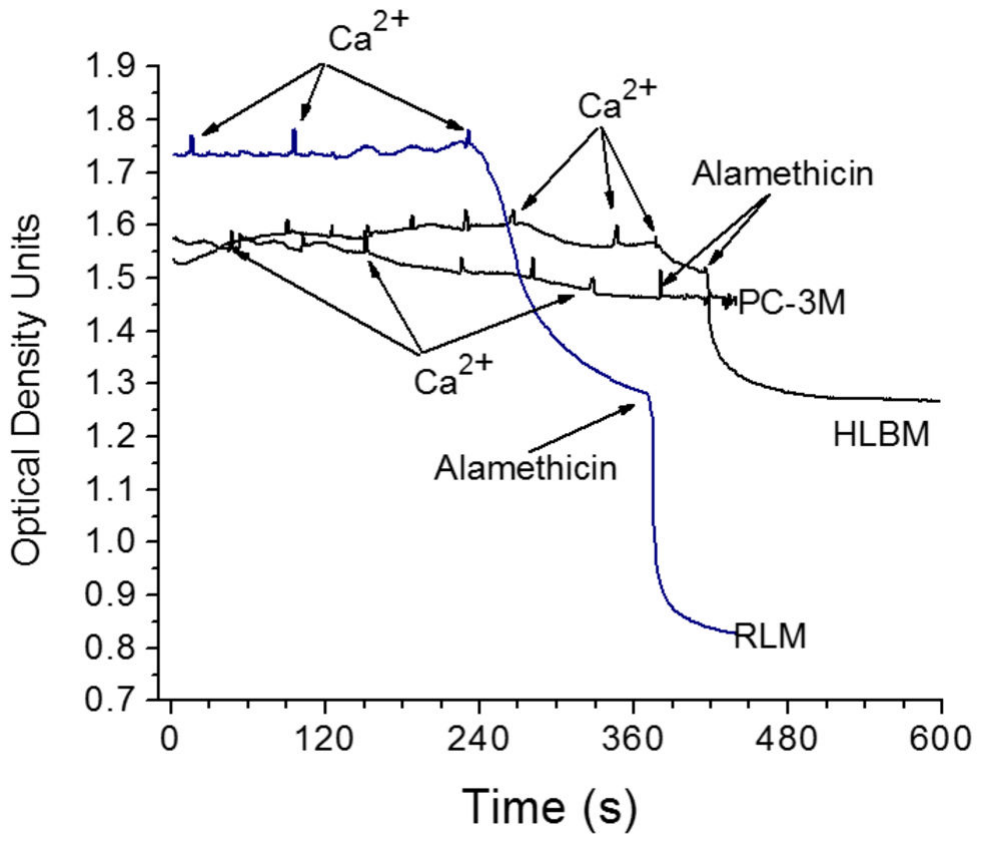
Swelling of mitochondria from rat liver, human lymphoblastoid and PC-3 cells. **Incubation conditions** (**Medium B**) sucrose 210 mM, KCl 20 mM, glycyl-glycine 3mM, pH 7.2, KH_2_PO_4_ 1 mM, succinate 10 mM, mitochondria 0.5 mg, final volume 1.0 ml. Mitochondrial swelling was recorded as optical density (OD) at 520 nm using Shimadzu Multispec-1501 model spectrophotometer. Additions: Ca^2+^ 50 nmol/ml, alamethicin 4 µg/ml.

For the LNCaP and PC-3 and to some extent for DU145 prostate cancer cell lines, the activities of Complex I, measured as the rate of reduction of DB, were high and showed a strikingly narrow DB concentration range for maximal rate of NADH oxidation (Figure 4A). By contrast, Complex I activity of SMP from PrEC, HFS, and HLB cells, were lower and showed a rather broad range of DB concentrations providing maximum activities, although the activities themselves differed significantly (Figure 4A, B). The SMP from the PrEC and HFS cells showed very low Complex I specific activities in comparison to SMP from HLB and the PC cells mitochondria. The large differences in the responses to changes in the DB concentration observed between the PC cell mitochondria (Figure 4A) and normal PrEC cells and the non-prostate cells (Figure 4B) may provide an indication of the differences in the lipid-protein interactions in the mitochondrial membranes, which could be responsible for the unusual lack of response of PC cell mitochondria upon addition Alamethicin, a pore forming peptide (see Figure 6).

Figure 5 shows a representative experiment of measurements of the Complex I kinetic parameters for oxidation of NADH in the presence of the optimal DB concentration shown in Figure 4. There were significant differences between SMP from the PC cells and other cell lines. SMP from the three prostate cancer cell lines had similar, relatively low affinity for NADH (K_M_^NADH^ = 20+2.5 µM). In comparison, the affinities for NADH were correspondingly 5, 4, and 11 fold higher than with the SMP from PrEC (K_M_^NADH^ = 4+0.5 µM), HT1080C (K_M_^NADH^ = 5+2 µM), and HLB (K_M_^NADH^ = 1.8+0.1 µM) cells. The low affinity for NADH could explain to some extent the relatively low rates of the State 3 oxidation of the NAD-dependent substrates by the PC cells mitochondria (see Figure 2).

The values of V_MAX_ for NADH oxidation by Complex I were also strikingly different between SMP from the prostate cancer cells and PrEC cells. V_MAX_ reflects to a large extent the amount of an enzyme present in the system under study [59]. The V_MAX_ values for the Complex I were 0.3-0.45 mM DB reduced/min/mg protein for the prostate cancer cells, and 0.028 mM DB reduced/min/mg protein for the PrEC SMP, a 10 to 16-fold difference. Thus PrEC cells not only had fewer mitochondria, but the mitochondria also had much lower content of Complex I per mitochondrion than mitochondria from the metastatic prostate cancer cells.

### Ca^2+^-induced permeability transition of PC-3 and HLB mitochondria

For a long time, most of our knowledge on the Ca^2+^-dependent permeability transition of mitochondria was based almost exclusively on experiments with the rat liver mitochondria (RLM) [60]. Large amplitude osmotic swelling of RLM was considered as a classical manifestation of permeability transition pore opening induced by calcium in the presence of inorganic phosphate (CaPi) (see Figure 6). However, we found that unlike RLM, mitochondria from other tissues may not undergo swelling [28], and even depolarization of mitochondria during calcium sequestration is not indicative of permeability transition [50]. We show that mitochondria from the HLB and PC-3 cells did not undergo large amplitude swelling during Ca^2+^ loads (Figure 6). HLB mitochondria did undergo swelling upon addition of alamethicin, a bacterial nonspecific pore forming antibiotic, while alamethicin was not effective with the PC-3 mitochondria (Figure 6). Thus large amplitude swelling of mitochondria cannot be used with the HLB and PC-3 mitochondria as an indication of opening of the permeability transition pore. Therefore, we used other methods to register permeability transition in these mitochondria.

As mentioned in the Methods, we have restricted assay of permeability transition to mitochondria from the HLB cells that can be grown in the rotary bottles, and the PrEC and PC-3 cells that can be harvested without treatment of cells with a protease. The reason for this lies in our observation that treatment of a tissue (skeletal muscle, heart muscle, liver) before homogenization, or isolated mitochondria with Nagarase or Tripsin significantly increased the mitochondrial calcium retention capacity (Panov A, unpublished data).

**Figure 7:**
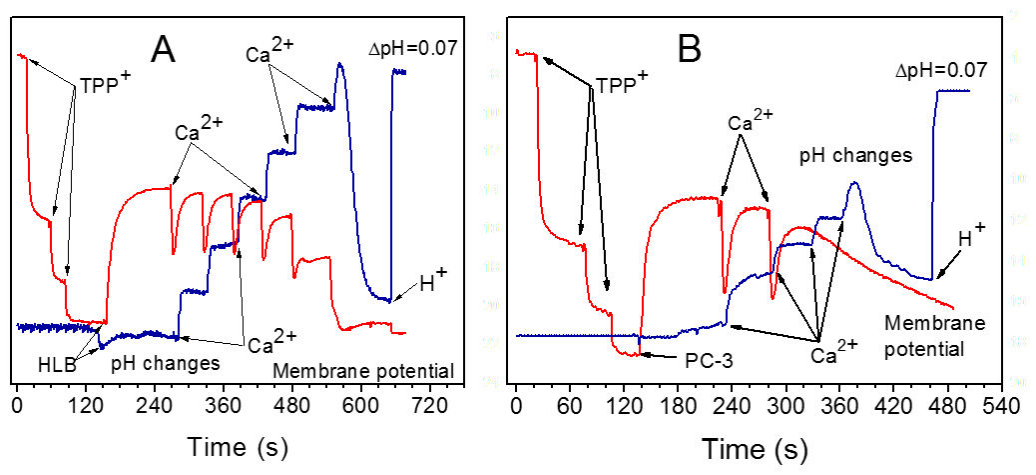
Changes in the membrane potential (red) and medium pH (blue) during titration of mitochondria with calcium. (**A**) Human lymphoblast mitochondria. (**B**) Mitochondria from PC-3 prostate cancer cells. **Additions**: TPP^+^ was added in 0.5 µM aliquots, final concentration 1.5 µM; Ca^2+^ 20 nmol/ml, HCl 125 nmol/ml caused ΔpH of 0.07.

**Figure 8:**
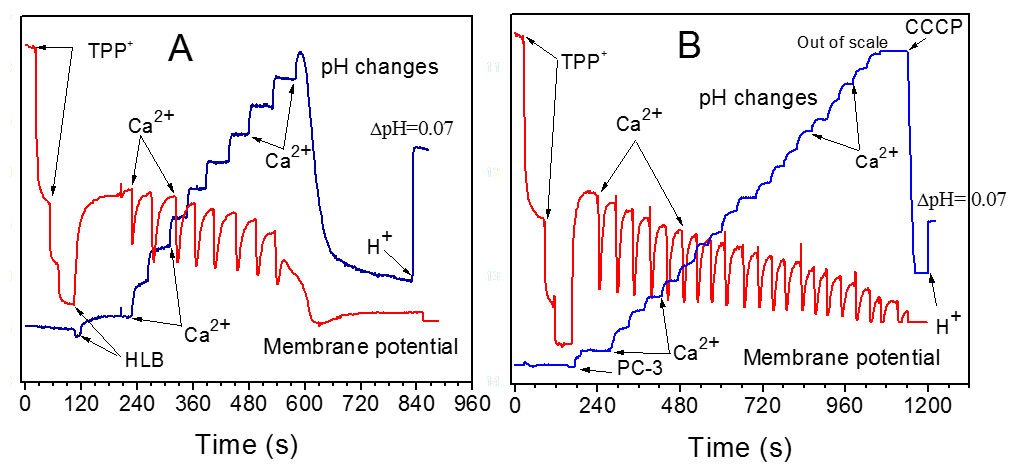
Effect of cyclosporine A on changes in membrane potential (red) and medium pH (blue) during titration of mitochondria with calcium. (**A**) Human lymphoblast mitochondria. (**B**) Mitochondria from PC-3 prostate cancer cells. Incubation conditions as in Figure 8, except that 0.5 µM CsA was present. **Additions**: TPP^+^ was added in 0.5 µM aliquots, final concentration 1.5 µM; Ca^2+^ 20 nmol/ml, CCCP 0.5 µM, HCl 125 nmol/ml caused ΔpH of 0.07.

Figures 7 and 8 show changes of membrane potential and the medium’s pH during gradual Ca^2+^ loading of the unprotected HLB and PC-3 mitochondria oxidizing succinate (Figure 7), and the mitochondria protected by 0.5 µM cyclosporine A (Figure 8). As mitochondria consume added Ca^2+^ via the electrogenic calcium uniporter, the ΔΨ dropped, and was then restored to the initial level. As more and more Ca^2+^ was added, the electrogenic cycling of calcium also increased and this was responsible for gradual decline in ΔΨ. When the mitochondrial permeability transition pore (mPTP) opened, ΔΨ collapsed almost instantly with the HLB mitochondria and very slowly with the PC-3 cell mitochondria. The pH method of registration of Ca^2+^ consumption by mitochondria allows precisely registering the moment of the mPTP opening. The pH method is the most reliable for quantitative evaluation of the amount of Ca^2+^ consumed by the mitochondria before the permeability transition occurs – the calcium retention capacity (CRC) [28,50]. When mPTP opened, the alkalization of the incubation medium was associated with dissociation of calcium triphosphate (Ca_3_(PO_4_)_2_) in the matrix, and binding of H^+^ to the released PO_4_^3-^ anion to form HPO_4_^2-^ and H_2_PO_4_^-^ anions in accordance with the buffer’s pH [28,61]. Thus alkalization of the medium unequivocally marks the time of the PTP opening. We implemented simultaneous measurements of the ΔΨ and pH. However, it should be kept in mind that depolarization of mitochondria is not always associated with the opening of mPTP, while opening of mPTP always results in the collapse of ΔΨ [50,60]. This is illustrated in figures 7 and 8. In the experiment with HLB mitochondria (Figure 7), instant depolarization of mitochondria and alkalization of the medium occurred almost simultaneously due to the opening of the large pore.

**Figure 9:**
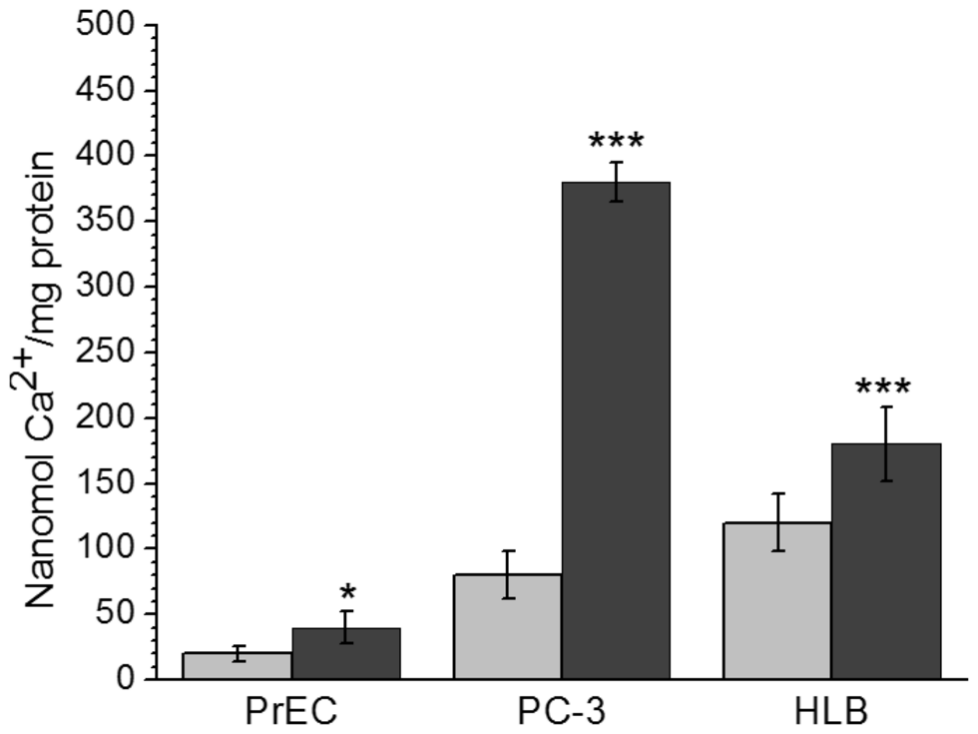
Calcium retention capacities of mitochondria from normal prostate PrEC, human lymphoblastoid (HLB) cells, and the prostate cancer PC-3 cells. **Incubation conditions** as in Figures 7-8. **Grey bars** – unprotected mitochondria oxidizing succinate, **dark bars** –mitochondria protected by Cyclosporin A 0.5 µM + oligomycin 2 µg/ml + ADP 50 µM. Data are M ± standard error calculated from 3 separate isolations of mitochondria. Data are expressed as nanomol Ca^2+^/mg mitochondrial protein. Statistics: * *p* < 0.1; *** *p* < 0.001. The data for protected by CsA mitochondria were compared with the corresponding unprotected mitochondria. The data for the PC-3 cell mitochondria were compared with those for the PrEC cell mitochondria.

Figure 8 shows responses to Ca^2+^ of the HLB (Figure 8A) and PC-3 (Figure 8B) mitochondria treated with cyclosporine A (CsA). CsA is the most powerful known inhibitor of permeability transition. Normally, CsA strongly delays the onset of mPT but does not prevent it, as illustrated in the case of mitochondria from HLB (compare Figures 7A and 8A) when CsA increased the CRC1.5 fold. However, it was not possible to induce mPTP opening by calcium loading of the PC-3 mitochondria protected with CsA (Figure 8B). Gradual decline of the signal of the TPP^+^-sensitive electrode more likely reflected the displacement of TPP^+^ from the matrix by accumulating calcium phosphate salts, rather than true depolarization [28]. The pH trace, shown in Figure 8B, demonstrates that unlike mitochondria from HLB, PC-3 mitochondria in the presence of CsA extruded protons unevenly, with varying H^+^/Ca^2+^ ratios. In comparison with the unprotected mitochondria, in the presence of CsA the PC-3 mitochondria were capable consuming very large amount of calcium without opening the mPTP. We could enable the Ca^2+^ release and opening of the mPTP only by addition of CCCP, a protonophore that stimulates mitochondrial depolarization. Before the addition of CCCP, the PC-3 mitochondria were capable to consume almost 5 times more Ca^2+^ in the presence of CsA (Figure 8B), than in the control experiment (Figure 7B). Thus, experiments presented in Figures 7 and 8 show that the PC-3 mitochondria have abnormal properties of the Ca^2+^-induced permeability transition. With the PrEC mitochondria, in the presence of CsA the CRC increased from 20 to only 40 nmol Ca^2+^/mg protein (Figure 9).

Figure 9 compares the calcium retention capacities of mitochondria from normal prostate PrEC cells, normal HLB cells and the prostate cancer PC-3 cells oxidizing succinate in the absence and in the presence of cyclosporine A. We have shown earlier that the CRCs of mitochondria isolated from the similar cell lines, but originating from different individuals, varied significantly [50]. Therefore, responses to Ca^2+^ and CsA of mitochondria from different cell lines can be evaluated only qualitatively. Figure 9 shows that unprotected PrEC mitochondria, had negligible ability to retain Ca^2+^ (about 20 nmol Ca^2+^/mg protein), whereas PC-3 mitochondria had a 4-fold higher resistance to calcium loads. In mitochondria protected with CsA, the CRC for PrEC mitochondria increased 2-fold, for HLB mitochondria the CRC increased by 50%, whereas for the PC-3 mitochondria the CRC increased almost 5-fold and was 9-fold higher than for the PrEC mitochondria. Importantly, unlike mitochondria from the PrEC and HLB cells, the CsA-protected PC-3 mitochondria did not open spontaneously permeability transition pore, but only after addition of the protonophore CCCP.

## Discussion

The cancerous and non-cancerous cells used in this study originated from different tissues and individuals [38,43,48,49]. Dissimilarities in culture conditions may also contribute to the observed differences in the metabolic parameters of the cells. Earlier we have found that mitochondria from the same cell type, namely human lymphoblastoid cells (HLBM), obtained from different individuals had quantitatively different yields per 1 gram of cells, respiratory activities and capacities to sequester calcium phosphate [50]. Qualitatively, normal HLBM were similar to each other and distinct from HLBM from patients with Huntington’s disease [50]. HLBM from Huntington’s disease patients also had quantitative dissimilarities in respiration rates and calcium retention capacities, but qualitatively they were uniquely similar to each other [50]. There was no possibility and sense for statistical comparison of HLBM from normal individuals and patients with Huntington’s disease. Therefore, interpretation of the data when comparing cells of different genetic background and culture conditions should be done with caution and based largely on qualitative properties. Quantitative comparisons can be done only within the same cell line.

The discussion of the results presented in this article, with respect to the papers published on the cancer energy metabolism topic, will be relatively limited. Firstly, this work so far is the only one performed on mitochondria isolated from normal and prostate cancer cell lines; and secondly, we considered it difficult discussing numerous publications on metabolic properties and the roles of mitochondria in prostate cancer cells cultured in the presence of antibiotics [21,23,36–38]. Aminoglycoside antibiotics (streptomycin, gentamicin) are toxic to mitochondria in several ways [39–41,62]. We repeatedly observed that mitochondria isolated from cells cultured in the presence of antibiotics (penicillin and streptomycin) do not respire on any substrate. Higgins et al. [38] cultured PC-3, LNCaP and DU145 cells in the presence of 1% penicillin-streptomycin and concluded that mitochondria in these cells were dysfunctional. Therefore, publications where cells were cultured in the presence of antibiotics are at least confusing and difficult for interpretation. Because of this, many of the reviewing papers are also confusing.

A comparison of the results obtained with mitochondria isolated from cultured cells in the absence of antibiotics revealed important metabolic features that are common only for the prostate cancer cell mitochondria. In general, the data presented in the current study support the bioenergetic hypothesis of prostate cancer, and the important role of mitochondria in the loss of the apoptotic potential by metastatic prostate cancer cells [6–8,32,44,63].

### Metabolic Features of Mitochondria from Normal and Cancerous Prostate Cells

Normal prostate epithelial cells are characterized by a high level of citrate production [44]. This metabolic feature can be explained by the regulatory role of Zn^2+^ on the catalytic activity of the mitochondrial *cis*-aconitase. At physiological concentrations, Zn^2+^ acts as a potent competitive inhibitor of *cis*-aconitase in prostate mitochondria, and thus inhibits further transformation of citrate in the tricarboxylic acid cycle [44,64]. In addition, in prostate mitochondria aconitase occurs at low concentration [65]. This explains why respiration rates per gram of prostate tissue are generally considered to be notably low [44]. Our data presented in Figure 2 also show that the rates of citrate oxidation by the isolated mitochondria from normal cultured prostate cells (PrEC) were very low. Addition of ADP caused almost no acceleration of respiration; therefore the respiratory control ratio were also very low (RCR=1.2). Our experiments have also shown that mitochondria from PrEC cells oxidized succinate and glutamate at very low rates in all metabolic states as compared with the mitochondria from other cell lines studied (Figure 2). Thus, the low respiratory activity of normal prostate cells more likely was bound to relatively low content of mitochondria per cell (Figure 1) and lower contents of respiratory enzymes in the mitochondria.

Cancer transformation of prostate cells was accompanied by a several-fold increase in the number of mitochondria per cell (Figure 1 [42]) and the content of respiratory enzymes per mg of mitochondrion. Figure 2 shows that in comparison with the normal PrEC cells, mitochondria from prostate cancer PC-3, DU145 and LNCaP cells, increased the state 4 oxidation of citrate 19-, 10- and 4-fold correspondingly. However, PC cells’ mitochondria oxidizing citrate also failed to increase respiration upon addition of ADP, and therefore the respiratory control ratios were also low as with the PrEC mitochondria (Figure 2). This could suggest, that the state 4 respiration rate was increased due to increased content of respiratory enzymes, but further metabolism of citrate remained largely inhibited similar to PrEC mitochondria. This was one possibility. Alternatively, however, the sluggish response to ADP was also observed with the non-prostate cancer HT10780C mitochondria (Figure 2). With mitochondria from normal HLB cells oxidizing citrate the state 3 respiration was also lower than with succinate or glutamate. All these facts could favor the assumption that the poor response to ADP in the PC mitochondria was also associated with the fact that citrate is a powerful chelator of calcium [66]. It is well established that three intramitochondrial dehydrogenases, namely, pyruvate dehydrogenase complex, a-ketoglutarate dehydrogenase complex, and the NAD-linked isocitrate dehydrogenase, show significant activation by small increases of Ca^2+^ in the matrix [67,68]. However, the high rates of the State 3 citrate oxidation and RCR > 4 with the noncancerous HLB mitochondria argue against this assumption (Figure 2). Thus, we can suggest that in in the prostate cancer cell mitochondria, in spite of the several-fold increase in the content of respiratory enzymes (Figure 2), oxidation of citrate cannot function normally via the “classical” tricarboxylic acid cycle.

This suggestion agrees with our finding that in the submitochondrial particles prepared from the isolated PC cell mitochondria, Complex I had low affinity for NADH (Figure 5). In addition, a very high electrogenic component (ΔΨ) of the proton motive force (Δμ_H+_) (Figure 3) in the PC mitochondria also implies that these mitochondria utilize glutamate, the only electrogenically transported mitochondrial substrate. In our calculations of ΔΨ we suggested to account the volume of the matrix space for prostate mitochondria as 1 µl/mg protein, which was a large overestimation. Figure 6 shows that PC-3 mitochondria (as well as mitochondria from DU145 and LNCaP, data not shown) did not undergo large amplitude swelling even in the presence of alamethicin, a bacterial pore-forming peptide. This suggests that the volume of the matrix space in PC cells mitochondria may be less than 1 µl/mg, and thus the actual values for ΔΨ were higher than 200 mV. This implies that prostate cancer cell mitochondria have lower values of ∆pH, which is the driving force for mitochondrial transport of most respiratory substrates [69]. Thus, high ΔΨ, will promote electrogenic transport of glutamate in exchange for aspartate [70], and further oxidation of glutamate via the conversion to α-ketoglutarate in transaminase reactions [71].

**Figure 10:**
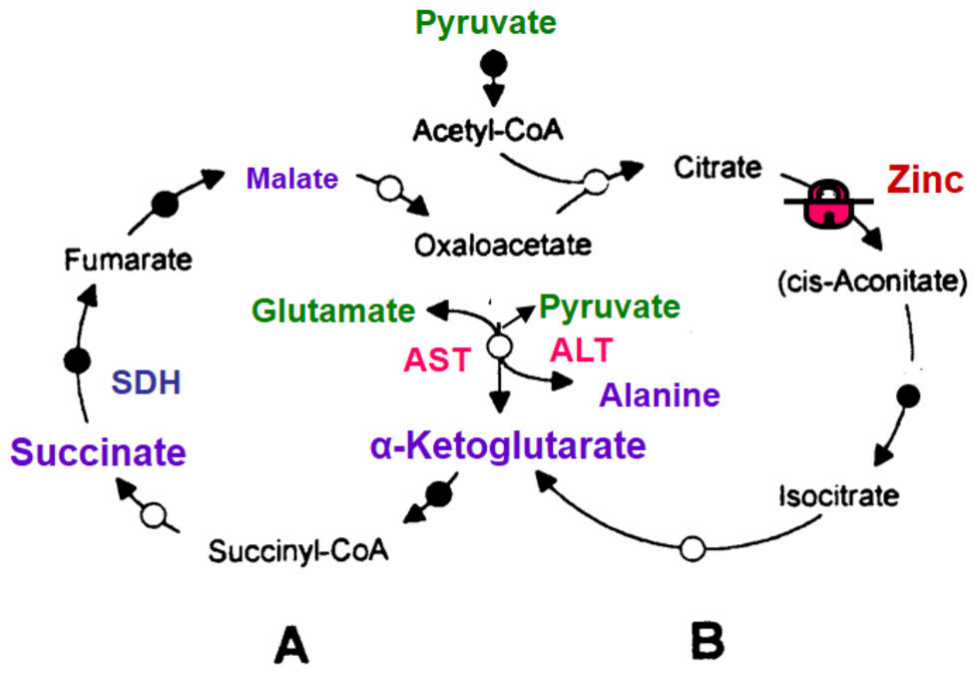
The Tricarboxylic Acid Cycle in the presence of pyruvate and glutamate operates as two coupled cycles A and B. In the presence of glutamate and/or pyruvate as substrates, the high activities of AST and ALT may “short-circuit” oxaloacetate and α-ketoglutarate, thus converting the “normal” TCA cycle into two independently operating cycles A and B [71]. Malonate is a quasi-irreversible inhibitor of SDH, whereas oxaloacetate (OAA) is a competitive but strong inhibitor. SDH has similar affinities for succinate and malate, but oxidation of malate results in the formation of OAA on the enzyme’s active center, which makes malate a strong inhibitor of SDH. Cycle B in prostate cells is inhibited by Zn^2+^ at the level of *cis*-aconitase. **Abbreviations**: ALT – alanine aminotransferase; AST –aspartate aminotransferase; SDH – succinate dehydrogenase (Complex II).

Taken together, the above features of respiration of the prostate cancer cell mitochondria suggest alternative functioning of the TCA cycle (Figure 10). There is evidence that in the presence of glutamate + malate, the TCA cycle may operate as two coupled cycles: one (cycle A) leads from α-ketoglutarate to oxaloacetate, and another (cycle B) from oxaloacetate to a-ketoglutarate that includes the citrate synthase reaction (Figure 10) [45,71]. In the presence of glutamate and/or pyruvate high activities of aspartate aminotransferase and alanine aminotransferase may “short-circuit” oxaloacetate and α-ketoglutarate, thus converting the “classical” TCA cycle into two independently operating cycles A and B. According to Yudkoff et al. [71], the flux of substrates through cycle A may be 3-5-fold faster than that through the cycle B. In prostate mitochondria inhibition of *cis*-aconitase by Zn^2+^ causes accumulation of citrate, which is produced in cycle B from acetyl-coA and oxaloacetate. Acetyl-CoA may be derived from pyruvate or fatty acids, and oxaloacetate, which is produced in the cycle A. Thus, under conditions of high ΔΨ, glutamate, and hence glutamine, may become the main substrates for the prostate cancer cell mitochondria. Glutamate is transaminated to α-ketoglutarate, which is further metabolized to succinate and oxidized by prostate cancer cell mitochondria at high rates in the State 3 and State 3U (Figure 2).

High activity of electrogenic transport of glutamate in cancer mitochondria with high ΔΨ might explain the mechanism of development of cachexia in some metastatic cancers: tumor mitochondria deprive other tissues of glutamate and glutamine, and thus cause degeneration of the tissues. This agrees with the recently suggested hypothesis that cancer cells behave as “parasitic organisms” [72].

### Antiapoptotic properties of prostate cancer cell mitochondria

It has been shown that apoptotic activity in the primary androgen independent prostate carcinomas was significantly lower than in the matched androgen dependent prostate carcinomas while the proliferative activity remained unaffected [6–8]. Mitochondria play a key role in regulation and initiation of apoptosis by several mechanisms, of which opening of the Ca^2+^-dependent mitochondrial permeability transition pore (mPTP) is the decisive event determining the fate of a cell [9–11]. It has been found that diminished Complex I activity can directly inhibit opening of mPTP [26,27]. High ΔΨ also prevented opening of the permeability transition pore in mitochondria (see for review, 60), thereby preventing apoptosis [53,73]. Higher than normal mitochondrial electrical membrane potential (ΔΨ) has long been considered a common feature of cancer cells (reviewed in 52). However, most of the data were obtained using fluorescent dyes on whole cells [52], which give only qualitative evaluation of ΔΨ, and interpretation of the data can be further complicated by the cell’s multidrug resistance protein [74]. Figure 3 presents the results of the direct measurements of ΔΨ in isolated mitochondria using a TPP^+^-sensitive electrode and specific constants for binding TPP^+^. Importantly, the prostate cancer cells mitochondria had 20-30 mV higher ΔΨ values as compared with mitochondria from the control human PrEC and HLB cells, and the non-prostate human fibrosarcoma HT1080C cells. The difference was quite significant because relationship between ΔΨ and mitochondrial proton permeability is non-linear [75], and the higher ΔΨ means that the conductance of the inner mitochondrial membrane for protons was lower than normal. As we suggested earlier, in realty the values of ΔΨ in PC mitochondria can be higher than -200 mV.

The mechanisms by which prostate cancer cell mitochondria acquire abnormally high ΔΨ remain unknown. One possibility is that the high ΔΨ results from a low nonspecific proton leak through the inner mitochondrial membrane due to high cholesterol content in the membrane [45]. Another possibility presumes that cancer transformation can modify specific channel for protons. The adenine nucleotide translocase (ANT) was shown to be a site of regulation of the specific permeability for H^+^ and K^+^ ions [76], and thus control opening of the permeability transition pore [28]. Torroni et al. [77] reported that neoplastic transformation of cells was associated with a shift in the isoenzyme pattern of the ATP/ADP carrier. Both Bcl-xL and Bcl-2, antiapoptotic members of the Bcl family, were found in prostate cancer cell lines [23]. It has been shown that the functional target of Bcl-2 is regulation of ΔΨ by enhancing H^+^ efflux from mitochondria, thus increasing the electrical component (ΔΨ) of the total electrochemical gradient (Δμ_H_^+^), which has a maximum of 250 mV [78].

High rates of respiration are also a necessary condition for sequestration of calcium as a quasi-insoluble calcium triphosphate salt, and thus prevent opening of mPTP and initiation of apoptosis. Figure 9 shows that as a result of metabolic changes in the prostate cancer mitochondria, the amount of Ca^2+^ necessary to initiate opening of the mPTP in unprotected mitochondria increased 4 times as compared with the normal PrEC mitochondria, and 10-fold in the presence of cyclosporine A. Thus mitochondria in the metastatic prostate cancer cells might have several mechanisms responsible for exceptionally high resistance to the Ca^2+^-dependent apoptotic cell death. Bagetto [45] suggested that cancer transformation is associated with a complex of non-random phenotypic changes, initiated by expression of oncogenes.

Taken together, the data presented in this paper demonstrate that due to complex transformations of the metabolic features of mitochondria, the metastatic prostate cancer cells become more resistant to apoptotic stimuli than normal prostate epithelial cells. These qualities of the prostate cancer cells can be the target for pharmacological interventions.

## Materials and Methods

### Cell lines and cell cultures

Normal human prostate epithelial cells, PrEC, were obtained from Clonetics Corporation, San Diego, CA. PrEC were grown in a serum-free PrEGM medium with supplements provided by Clonetics Corp. The established human prostate cancer cell lines, LNCaP [43], PC-3 [48] and DU145 [49], were obtained from the American Type Culture Collection, Rockville, MD. HT1080 clone c (HT1080C) cells were originally obtained from Dr. Carlo Croce (79).

All cancer cell lines were cultured in RPMI 1640 medium supplemented with 10% FBS (Gibco BRL, Life Technologies) at 37^o^C in a 95% air: 5% CO_2_ atmosphere. The cell lines were passaged twice a week, and the medium was changed every other day. Human lymphoblastoid cells (HLB) from healthy individual volunteers were prepared by infection of peripheral blood leukocytes with Epstein–Barr virus, and grown in RPMI 1640 with 10% FBS in rotary bottles. No antibiotics were used during culturing of the cells.

All cell lines were shown to be mycoplasma free by the Boehringer Mannheim BM-Cyclin test. The cells, grown in T225 flasks, were harvested at 60-65% confluence using 0.05% Trypsin, 0.5 mM EDTA, and washed in a medium containing 250 mM sucrose, 1 mM EGTA, 10 mM MOPS, pH 7.2. PrEC and PC-3 cells were harvested by shaking the flasks.

For mitochondria isolation, cells were harvested from 8–16 T225 flasks or 2 L rotary bottles.

### Isolation of mitochondria from the cells

The method of isolating mitochondria by using permeabilization of cells with digitonin [5] was not optimal for prostate cancer cell lines. In comparison with HLB and HT1080 cell, all PC cells showed a much higher resistance to the permeabilizing effect of digitonin, with the PC-3 cells being the most resistant. Even in the presence of the 4-fold higher concentration of digitonin suggested in [5] only about 60% of cells could be destroyed by homogenization. Therefore, we isolated mitochondria by swelling the cells in a mildly hypotonic buffer (120 mOsm) as described in [50]. The method was specifically designed for prostate cancer cells, and takes advantage of the fact that at a tonicity of 120 mOsm the whole cells swell but the mitochondria undergo only a low-amplitude swelling and have even higher ATP level and ΔΨ than mitochondria incubated under normal tonicity [80]. Consequently, the swollen cells were easily disrupted using the Wheaton Dounce Tissue Grinder, while mitochondria remained intact. The cells were washed twice with the buffer containing 250 mM sucrose, 1 mM EGTA, and 10 mM MOPS (pH 7.2). The cell’s pellets were weighed, and resuspended in 5 ml per 1 gram of cells of the low tonicity buffer containing sucrose 100 mM, MOPS 10 mM, pH 7.2, EGTA 1 mM, BSA 0.1%. The swelling of cells was controlled under microscope and, if necessary, water was added in small aliquots until all cells became swollen. The cells were allowed to swell for 2-5 minutes, and then homogenized with 20-25 sharp strokes of the tight pestle in a Wheaton Dounce Tissue Grinder. An aliquot of 1.25 M sucrose was added to bring the medium’s tonicity to 250 mOsm. The volume of the suspension was then tripled with the isolation buffer containing mannitol 210 mM, sucrose 70 mM, MOPS 10 mM (pH 7.2), EGTA 1 mM, 0.1% BSA, and centrifuged at 800 g for 5 min in a Beckman Avanty J-25 model refrigerated centrifuge, using a J25.5 rotor, at 4^o^C. It should be kept in mind that in cultured cells mitochondria are elongate and reticulated, and therefore elimination of nuclei and cell’s debris should be made with caution and adjusted for each cells line to obtain maximal yields of mitochondria. The procedure for isolation of mitochondria from the cultured cells was described in details [82]. Isolation of mitochondria using the hypotonic swelling procedure described above gave consistently higher yields of mitochondria per gram of cells and better functional properties of mitochondria than the isolation procedure with digitonin [5].

The mitochondrial protein concentration was determined using the Coomassie Protein Assay Reagent Kit (Pierce) and corrected for the BSA concentration in the isolation buffer.

### Polarographic analysis of mitochondrial respiration

Oxygen consumption by mitochondria was measured as described previously [81]. The following incubation medium (Medium A) was used: KCl 125 mM, MOPS 10 mM, pH 7.2, MgCl_2_ 2 mM, KH_2_PO_4_ 2 mM, NaCl 10 mM, EGTA 1 mM, CaCl_2_ 0.7 mM. Substrates were added at the following concentrations: succinate 10 mM without rotenone, glutamate 10 mM + malate 2 mM, citrate 10 mM + malate 2 mM. To ensure the reliable measurements of membrane potential and respiration rates, we added mitochondrial protein at 0.5 mg/ml, except mitochondria from the PrEC cells, which were used at 1.0 mg/ml because of the extremely low rates of respiration. Oxidative phosphorylation (State 3) was initiated by addition of 150 µM ADP. The uncoupled respiration (State 3U) was stimulated by addition of 0.5 µM cyanide-*m*-chlorophenylhydrazone (CCCP).

### Measurements of ΔΨ of the cell mitochondria

The electrical membrane potential (ΔΨ) was measured using the custom made tetraphenylphosphonium (TPP^+^)-sensitive electrode, as described previously [28,81].

### Estimation of the mitochondrial Inner Binding Constant (IBC) for TPP^+^

The precise calculations of mitochondrial ΔΨ values determined with the TPP^+^-sensitive electrode strongly depend on binding of TPP^+^ inside mitochondria with a negligible influence of binding to the outer compartments [55]. Therefore, we estimated the mitochondrial IBC for the cells under study using the H^3^ labeled TPP^+^ as described in [56]. For the PrEC and the three prostate cancer cell lines, the IBS values were close to each other and an averaged IBC = 13.1+1 was used in the calculations. For the HLB mitochondria the IBS = 15.2+1.5, for the HT1080C mitochondria the IBS = 10+0.8. We encountered problems in reliable determination of the mitochondrial matrix volume. Presumably, this was associated with the fact that mitochondria from all cell lines did not undergo swelling upon loads with calcium (see Figure 8), and thus, probably, had limited osmotically active volume. Therefore, we assumed that the volume of the mitochondrial matrix space was the same for all cell types (1 µl/mg mitochondrial protein). The suggestion was based also on the fact that at concentration of 0.3 mg/ml all mitochondria showed similar initial optical density. Additions of TPP^+^ at increments of 0.5 µM (final concentration 1.5 µM) served as the internal calibration for the membrane potential changes.

### Measurements of the mitochondrial Calcium Retention Capacity (CRC)

CRC is the amount of calcium (nmol Ca^2+^/mg protein) that can be loaded to mitochondria until the permeability transition occurs [28]. In our previous study of the permeability transition of the rat liver and skeletal muscle mitochondria we have found that treatment of mitochondria with proteases (Nagarse, Trypsin) significantly increased CRC (Panov A, unpublished data). Therefore, we restricted our study of permeability transition to those mitochondria, namely HLB cells, which can grow in suspension, PC-3 and PrEC cells that could be harvested from the flasks by shaking the flask with a sharp blow of the rubber mallet.

Three different methods were used to register opening of the permeability transition pore, and thus estimate CRC [28]. 1) We have measured pH changes of the incubation medium during Ca^2+^ consumption and release by the mitochondria. The pH measurements were performed using Corning pH meter model 440 equipped with a Corning combination microelectrode. The addition of 125 nmol/mL H^+^ (as HCl) at the end of an experiment caused a ΔpH of 0.07 pH units and served as the internal calibration. 2) Depolarization of the mitochondria registered with a TPP^+^-sensitive electrode as described elsewhere [28]. ΔΨ and pH changes were registered simultaneously. 3) The volumetric changes in the mitochondria were recorded at 520 nm using Shimadzu multispec-1501 model spectrometer.

The mitochondrial CRC were measured in a medium containing (Medium B): sucrose 210 mM, KCl 20 mM, glycyl-glycine 3mM, pH 7.2, KH_2_PO_4_ 1 mM, succinate 10 mM, mitochodria 0.5 mg, final volume 1.0 ml. Calcium was added to mitochondria in 5 µl aliquots of 5 and 10 mM stock solutions of CaCl_2_ of very high purity (Sigma), as described previously [28].

### Measurements of mitochondrial swelling

Mitochondria 0.5 mg/ml were incubated in Medium B with 10 mM succinate under constant stirring. Final volume 1 ml. Mitochondrial swelling was recorded as optical density (OD) at 520 nm using Shimadzu Multispec-1501 model spectrophotometer. Additions: Ca^2+^ 50 nmol/ml, alamethicin 4 µg/ml.

### Preparation of submitochondrial particles (SMP) and measurements of the activity of Complex I

SMP were prepared from mitochondrial suspensions containing 1.5 mg/ml mitochondrial protein by sonication of a 0.5 ml aliquot with 6 pulses of 1 sec duration using microtip of the Branson Sonifier 450 model (Duty cycle 30, Output control 5). The sonication medium contained 250 mM sucrose, 10 mM MOPS, 0.5% BSA and 2 mM EDTA. Measurements of activities of the complex I, were performed as described elsewhere [5].

### Data acquisition

The data acquisition was performed using the 202A model of DC amplifier/filter, Warner Instrument Corp., DMA interface and software from C & L Company, Pennsylvania (www.fluorescence.com).

### Chemicals

Sucrose, mannitol, and other chemicals were from Sigma and were of molecular biology grade. All solutions were prepared using twice glass distilled water.

### Statistics

Initial inspection showed that results were normally distributed. Therefore, parametric statistical procedures were used. Data are presented as mean ± standard error of 3-5 separate experiments. Comparisons between two groups were made by unpaired *t*-test; p values < 0.05 were considered significant.
